# Two distinct pathways of RNA polymerase backtracking determine the requirement for the Trigger Loop during RNA hydrolysis

**DOI:** 10.1093/nar/gkab675

**Published:** 2021-08-07

**Authors:** Hamed Mosaei, Nikolay Zenkin

**Affiliations:** Centre for Bacterial Cell Biology, Biosciences Institute, Faculty of Medical Sciences, Newcastle University, Baddiley-Clark Building, Richardson Road, Newcastle Upon Tyne, NE2 4AX, UK; Centre for Bacterial Cell Biology, Biosciences Institute, Faculty of Medical Sciences, Newcastle University, Baddiley-Clark Building, Richardson Road, Newcastle Upon Tyne, NE2 4AX, UK

## Abstract

Transcribing RNA polymerase (RNAP) can fall into backtracking, phenomenon when the 3′ end of the transcript disengages from the template DNA. Backtracking is caused by sequences of the nucleic acids or by misincorporation of erroneous nucleotides. To resume productive elongation backtracked complexes have to be resolved through hydrolysis of RNA. There is currently no consensus on the mechanism of catalysis of this reaction by *Escherichia coli* RNAP. Here we used Salinamide A, that we found inhibits RNAP catalytic domain Trigger Loop (TL), to show that the TL is required for RNA cleavage during proofreading of misincorporation events but plays little role during cleavage in sequence-dependent backtracked complexes. Results reveal that backtracking caused by misincorporation is distinct from sequence-dependent backtracking, resulting in different conformations of the 3′ end of RNA within the active center. We show that the TL is required to transfer the 3′ end of misincorporated transcript from cleavage-inefficient ‘misincorporation site’ into the cleavage-efficient ‘backtracked site’, where hydrolysis takes place via transcript-assisted catalysis and is largely independent of the TL. These findings resolve the controversy surrounding mechanism of RNA hydrolysis by *E. coli* RNA polymerase and indicate that the TL role in RNA cleavage has diverged among bacteria.

## INTRODUCTION

Transcription by multi-subunit DNA-dependent RNA polymerase (RNAP) can be interrupted by backtracking of RNAP, a phenomenon when the 3′ end of RNA disengages from the template DNA and active center and RNAP shifts backward along the template (1). Backtracking can occur upon misincorporation events, when the non-cognate NMP at the 3′ end of RNA that is non-complementary to the base in the template DNA, forces RNAP into 1 base pair (bp) backtracked state (2,3). Misincorporated elongation complexes (ECs) may comprise up to 7% of all elongation complexes in the cell (4). Backtracking may also occur in non-misincorporated ECs where thermodynamics of sequences of the nucleic acids scaffold of the EC (5) or their recognition by RNAP core (6) favor backtracking. For example, weak RNA-DNA hybrid (A:U rich) at the 3′ end of the transcript may induce and/or stabilize backtracking. RNAP core can recognize some sequences of the RNA-DNA hybrid, which also may stabilize the backtracked state by slowing translocation of the EC (such sequence is used in the present study) (6).

Disengagement of the 3′ end of the transcript from the active site renders the complexes incompetent in NTP addition and thus causes a strong pause of transcription. Backtracking, if not resolved, frequently represents a dead-end event (1) and may cause ‘traffic jams’ of the trailing RNAPs thus impeding gene expression (7) and obstruct progression of the replication fork resulting in genome instability (8).

While sequence-dependent backtracking can be rescued for example through pushing of RNAP by coupled translation (9), misincorporated backtracked ECs can be rescued only by hydrolysis of the phosphodiester bond of RNA by RNAP active site. Hydrolysis generates a new 3′ terminus in line with active center and revives the EC making it ready to resume nucleotide addition (10). The hydrolysis of phosphodiester bonds of the transcript is catalyzed by the same two Mg^2+^ ions of the active center that participate in RNA synthesis and is assisted by the flipped out 3′ end nucleotide of the transcript (transcript-assisted RNA hydrolysis (3)). This 3′NMP participates in chelation of the weakly bound second Mg^2+^ and/or in a general acid-base catalysis, depending on the identity of the 3′NMP.

RNA hydrolysis by *Thermus aquaticus*, *Deinococcus radiodurans* and cyanbacterial RNAPs also heavily relies on the Trigger Loop (TL) (11–14), the catalytic domain of the RNAP active center (15). The TL can change between the catalytically active, folded, and catalytically inactive, unfolded, conformations (16). During nucleotide addition (or its reversal – pyrophosphorolysis), the TL folds to stabilize the transition state of the reaction by its H936 and R933 (here and after *E. coli* numbering) (17). During phosphodiester bond hydrolysis by *T. aquaticus* RNAP, the TL directly participates in the catalysis as a general base (through its H936) and by orienting the RNA 3′ NMP for transcript-assisted hydrolysis (11). Mutation of catalytic H936 or deletion of the TL in *T. aquaticus* RNAP led to >200-fold decrease in the rate of the reaction in either correct or misincorporated ECs (11).

However, the data on the role of the TL in intrinsic hydrolysis by *E. coli* RNAP turned out to be controversial. Our results indicated that H936A substitution in the TL of *E. coli* RNAP significantly (>30-fold) slows down proofreading in ECs that are stabilized in 1 or 2 bp backtracked states after misincorporation of erroneous NMPs (11). Similar results were obtained with *E. coli* RNAP lacking the TL; >100 fold reduction in rate of cleavage in EC with one or two mismatched 3′NMPs (13). In contrast, in the correct *E. coli* ECs (with 3′ end of RNA complementary to the template), the TL played little role in RNA hydrolysis (∼3 fold decrease in the rate of cleavage by H936Q mutant RNAP) (18). This study suggested that the TL assists backtracking and/or positions the disengaged from the template 3′ end of RNA but does not participate in the catalysis as a general base. It should be noted that in correct ECs the rates of backtracking and catalysis of RNA cleavage cannot be clearly separated, unless the EC is stabilized in the backtracked state in a sequence-dependent manner (6).

Salinamide A (SAL), the most extensively studied molecule of structurally related antibiotics salinamides, selectively inhibits bacterial RNAP. The crystal structure of *E. coli* RNAP holoenzyme in complex with SAL showed that SAL makes direct interactions with bridge-helix ‘cap’ (the fork loop, bridge helix N-terminal hinge and the link region) (19). The authors proposed that SAL functions by preventing conformational changes of the bridge-helix N-terminal hinge necessary for nucleotide addition, but not through disabling the TL.

Here, we show that SAL inhibits functions of the TL, and, using it as a molecular tool, demonstrate the existence of two distinct types of *E. coli* backtracked complexes, one efficient in RNA cleavage and another one not. The TL is required to convert the cleavage-inefficient into cleavage-efficient type of backtracked EC.

## MATERIALS AND METHODS

### Purification of SAL

*Streptomyces* sp. CNB091 was cultivated in shake flasks for 8 days at 30°C in 10 L of sea water-based media (10 g/l starch, 4 g/l yeast extract, 2 g/l peptone, 1 g/l CaCO_3_, 40 mg/l Fe_2_(SO_4_)_3_, 100 mg/l KBr; all dissolved in sea water). The cell mass was removed by centrifugation and supernatant subjected to 20 g of Amberlite XAD-16 resin (Sigma-Aldrich) pre-washed with deionized water. The material was eluted from the beads with 1 L of methanol. The extract was concentrated to aqueous residue *in vacuo* and the pH was adjusted to 7. Organic extraction was performed with ethyl acetate at equal volume followed by evaporation of organic phase to dryness under reduced pressure. The dried extract was dissolved in 2 ml 10% aqueous acetonitrile and subjected to a HyperSep™ C8 cartridge (Thermo Scientific) using a vacuum manifold (Thames Restek). SAL was eluted from the cartridge by 60% aqueous acetonitrile. The solvent was removed under reduced pressure; the material was dissolved in 20% aqueous acetonitrile and separated on C18 column (C18 ZORBAX Eclipse XDB-5 μm-9.4 × 250 mm, Agilent) in acetonitrile gradient. SAL containing fractions were evaporated under reduced pressure yielding 12 mg of SAL which was dissolved in 1 ml of methanol and stored at -20°C. For transcription reactions SAL was diluted in water.

### Purification of *E. coli* RNAP enzyme

*Escherichia coli* RNAP lacking the TL was constructed by substituting residues 931-1135 with Gly in the overexpression plasmid pVS14 (coding for *E. coli* RNAP subunits α, β and β’, with hexahistidine tag on C terminus of β’ subunit (20)) using HiFi DNA assembly cloning kit (New England Biolabs). Plasmids encoding for WT or mutant RNAPs were transformed along with pACYCDuet-1_Ec_rpoZ (encoding for ω subunit) in T7 Express strain (New England Biolabs). WT and ΔTL *E. coli* core RNAPs were expressed and purified as described (21), except for additional nickel-affinity chromatography that followed Heparin chromatography step.

### Transcription

**Figure 1.**
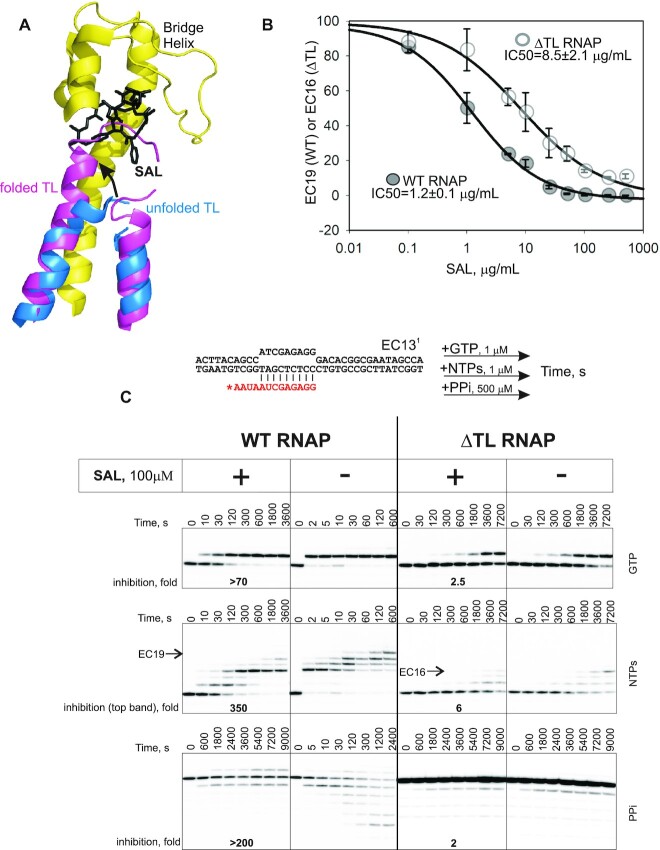
SAL inhibits RNAP through blocking functions of the TL. (**A**) Alignment of structures of RNAP with bound SAL and unfolded TL (PDB 4MEX) and RNAP with the folded TL (PDB: 4YLN). Note the clash of the folded TL with SAL molecule. (**B**) SAL IC50s were measured in inhibition of formation of EC19 and EC16 by WT and ΔTL RNAPs, respectively, during chase with all NTPs as in the middle of panel (C). Error bars are SD based on three replicates. IC50s are shown next to the corresponding plots (± is standard error). (**C**) Kinetics of single-nucleotide addition (GMP), chase in the presence of all NTPs and pyrophosphorolysis by WT and ΔTL RNAPs in the absence or presence of SAL. The scheme of the EC13^1^ is shown above the gels (asterisk depicts radiolabel at the 5′ end). Folds of inhibition by SAL are shown at the bottom of the gels. Minimal possible inhibition for GMP incorporation is depicted as we could not quantify accurately the rate of reaction without SAL. During chase, EC19 (WT RNAP) and EC16 (ΔTL RNAP) were used for quantification. Note that due to reincorporation of NTPs that are released during pyrophosphorolysis, the approximate rate of pyrophosphorolysis was determined at initial time points, when NMP incorporation was minimal (see also main text).

**Figure 2.**
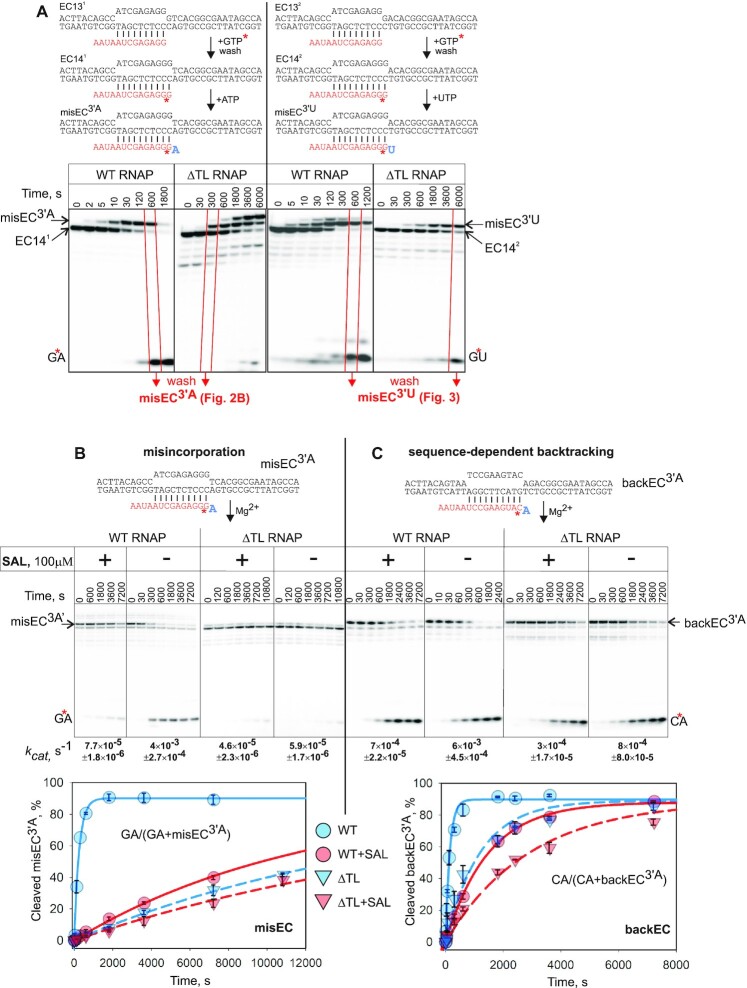
Two distinct types of backtracked ECs and role of the TL in conversion between them. (**A**) Formation of misincorporated ECs misEC^3′A^ and misEC^3′U^. Asterisk depicts radiolabel introduced during walking. Red lines depict the time points that were used to isolate (through washing) misEC^3′A^ and misEC^3′U^ for experiments shown in panel (B), and Figure 3. (**B** and **C**) Second phosphodiester bond hydrolysis by WT and ΔTL RNAPs in the presence or absence of SAL in ECs shown above the gels. misEC^3′A^ was obtained by misincorporation (see panel A), and backEC^3′A^ by walking to a sequence that causes stable 1 bp backtracking (asterisk depicts radiolabel introduced during walking). Cleavage was quantified as shown in the plot areas. Note that cleavage in EC14 that contaminated misEC^3′A^ and backEC^3′A^ was negligible for both, WT and ΔTL RNAPs, and did not affect quantification. Results were fitted in single exponential equation, and fits are shown below gels (error bars are SD based on three replicates). Reaction rates are shown below the gels (± is standard error).

ECs were assembled in 15 μl total volume. One pmol of template DNA (IDT) was incubated with 2 pmol of RNA (IDT) for 5 min at 45°C in transcription buffer (TB; 20 mM Tris-HCl, pH 8, 40 mM KCl), followed by cooling down at 4°C for 20 min. One pmol of WT or mutant RNAP was added and incubated at room temperature for 10 min. The complexes were then incubated with 10 pmols of non-template DNA bearing a 5′biotin tag for 5 mins at 37°C. The complexes were then immobilized on 5 μl of streptavidin beads slurry and washed first with TB containing high salt (1 M KCl) and then with TB. For Figure 1C, RNA of EC13^1^ was 5′ radiolabeled by T4 Polynucleotide Kinase and γ-[^32^P]-ATP prior to complex assembly, as described (17). SAL and reactants (concentrations shown in Figure 1B,C) were then added and reactions were activated with 10 mM Mg^2+^ at 37°C and allowed to proceed for times indicated or for 10 min (WT) and 2 h (ΔTL), respectively, for IC50 determination. RNA in misECs and backEC was radiolabeled by incorporation of α-[^32^P]-NMP (Hartmann Analytics) during walking of EC (see schemes in figures). The backEC^3′A^ was obtained by supplying EC13^2^ with 1 μM α-[^32^P]-CTP (3000mCi/mmol) and 10 μM ATP in the presence of 10 mM Mg^2+^ at 37°C for 15 min. To obtain misincorporated ECs (misEC^3′A^ and misEC^3′U^), corresponding EC13s were supplied with 1 μM α-[^32^P]-GTP (3000 mCi/mmol) and 1 mM (WT RNAP) or 10 mM (ΔTL RNAP) ATP or UTP. Reactions were initiated by addition of 10 mM Mg^2+^ for WT RNAP or 10 mM Mg^2+^/Mn^2+^ mixture for ΔTL RNAP and were carried out at 37°C for times indicated in Figure 2A. Prior the start of cleavage reactions, the unincorporated substrates and Me^2+^ were removed through washing with TB. 100 μg/ml SAL was added prior the cleavage reactions and they were activated with 10 mM Mg^2+^ at 37°C. The reactions were stopped after times indicated by addition of an equal volume of formamide-containing loading buffer. Products were resolved on 23% (20:3) denaturing polyacrylamide gel containing 8 M urea, revealed by PhosphorImaging (Cytiva) and analyzed using the ImageQuant software (Cytiva). Kinetic data were fitted using nonlinear regression in SigmaPlot. All experiments were repeated at least three times.

## RESULTS

### Inhibition of RNAP by SAL is dependent on TL

Crystal structure of SAL bound to RNAP showed that it should sterically prevent folding of the TL (19) (Figure 1A). To test whether SAL indeed inhibits functions of the TL, we constructed a mutant *E. coli* RNAP lacking the entire TL (ΔTL, β’931-1135). Transcription elongation complexes were assembled with fully complementary template and non-template DNA strands, 13 nucleotide-long synthetic RNA (scheme in Figure 1C) and either wild-type (WT) or ΔTL *E. coli* RNAP (EC13^1^) (6,22). SAL was added to saturating concentration of 100 μg/ml (IC50s were 1.2 ± 0.1 μg/ml and 8.5 ± 2.1 μg/ml for WT and ΔTL RNAPs, respectively; Figure 1B). As seen from Figure 1C (top panel), SAL strongly inhibited addition of a single NMP by WT RNAP. We could not accurately quantify the rate of reaction without SAL because the reaction was over in the first time point used (2 s) even in low GTP concentrations (1 μM), but it showed minimum a 70-fold inhibition by SAL. As expected ΔTL RNAP was much slower than WT RNAP in NMP incorporation. However, consistently with the prediction that SAL may block TL folding, NMP addition by ΔTL RNAP was only marginally affected by SAL (2.5-fold inhibition) (Figure 1C, top panel). Quantification of synthesis of 19 nucleotide-long transcript by WT RNAP and 16 nucleotide-long transcript by ΔTL RNAP (the longest visible RNAs; Figure 1C, middle panel) in the presence of all NTPs showed 340-fold inhibition of WT RNAP and only 6-fold inhibition of ΔTL RNAP by SAL. Note that inhibition of WT RNAP by SAL shows a uniform pattern throughout the template, with no obvious preference toward particular ECs, indicating that SAL inhibition is not similar to Tagetitoxin, inhibitor that binds in the vicinity of SAL-binding site and inhibits translocation of RNAP (22). Pyrophosphorolysis by WT RNAP was strongly inhibited by SAL (Figure 1C, bottom panel). Note that pyrophosphorolysis results in formation of NTPs, which are reincorporated back into RNA (even extend RNA further than initial EC). Therefore, an approximate 200-fold inhibition of pyrophosphorolysis from EC13^1^ by SAL was calculated based on the initial time points, when the reversal of pyrophosphorolysis (NMP incorporation) has not yet become significant. Also, as can be seen, WT RNAP managed to processively pyrophosphorolyze to EC8^1^, while in the presence of SAL processive pyrophosphorolysis further than EC12^1^ was completely blocked. Although ΔTL RNAP failed to perform processive pyrophosphorolysis beyond EC12^1^, SAL had only little effect even on the rate of pyrophosphorolysis in EC13^1^ (also determined based on initial time points; Figure 1C, bottom panel).

The results indicate that SAL inhibits phosphoryl transfer reactions by *E. coli* RNAP and this inhibition is linked to the functions of the TL. Taken together with the structural prediction (Figure 1A), data suggest that SAL prevents folding of the TL into catalytically active conformation.

### TL is required for transcript hydrolysis in misincorporated ECs

**Figure 3.**
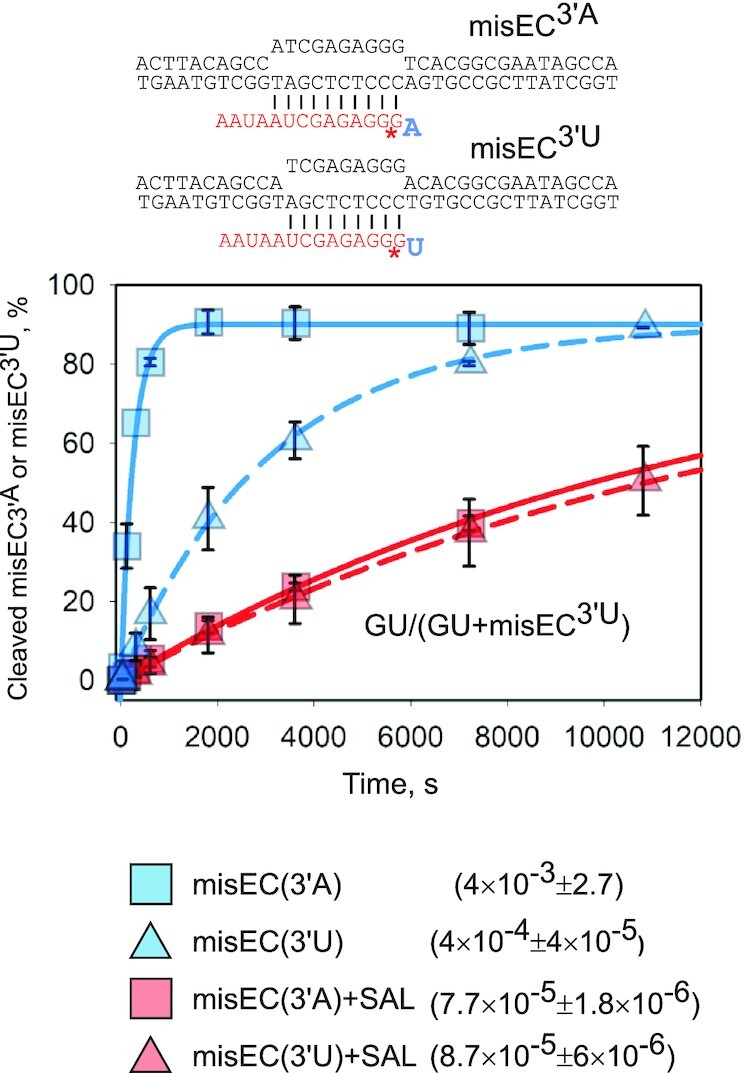
The TL changes coordination of misincorporated NMP for efficient transcript-assisted RNA hydrolysis. Kinetics of second phosphodiester bond hydrolysis in misincorporated ECs with erroneous 3′AMP and 3′UMP of the transcript in the presence or absence of SAL. misEC^3′A^ and misEC^3′A^ were formed and isolated as shown in Figure 2A. Asterisk in the schemes depicts radiolabel. Plots are single exponential fits of the data (error bars are SD based on three replicates). Rates of the reactions are shown in the legend (± is standard error).

We used SAL and ΔTL RNAP to investigate the discrepancy in involvement of the TL in phosphodiester bond hydrolysis. Since hydrolysis takes place in the backtracked state of the EC, the rate of reaction depends on the kinetics of backtracking. To exclude this influence, we used EC that was stabilized in the 1 bp backtracked state due to AMP at the 3′ end of RNA non-complementary to the template base. To avoid possible artefacts during direct assembly, such mismatched EC was prepared by natural misincorporation of a non-complementary AMP (misEC^3′A^; Figure 2A). Note that misincorporation in EC14^1^ by WT RNAP was rapidly followed by proofreading reaction, while ΔTL RNAP continued misincorporation to the following position. Therefore, when preparing misEC^3′A^, we chose time points when misEC^3′A^ was less contaminated by other ECs (red frames in Figure 2A). Reactions at these time points were washed and used in analysis of RNA hydrolysis (Figures 2B, 3). Note that hydrolysis in misEC^3′A^ releases exclusively GA dinucleotide. Also, in the polyacrylamide gels used, RNAs of other ECs or their possible cleavage products (which were negligible) would be separated from bands of misEC^3′A^ and GA, and would not affect quantification. Accordingly, during quantification we used only bands of misEC^3′A^ and GA (formula in the plot area in Figure 2B).

SAL strongly inhibited RNA hydrolysis in misEC^3′A^ (52 fold), suggesting the involvement of the TL in the reaction (Figure 2B). Indeed, the rate of hydrolysis by ΔTL RNAP was the same as that of WT RNAP with SAL (Figure 2B). Furthermore, SAL had no effect on the rate of the reaction by ΔTL RNAP (Figure 2B). We conclude that the TL is indeed required for phosphodiester bond hydrolysis in misincorporated EC.

Rate of phosphodiester bond hydrolysis depends on the identity of the 3′NMP of RNA, which assists catalysis by the RNAP active center (3,11). The reaction assisted by 3′AMP (misEC^3′A^) is 10 times faster than reaction assisted by 3′UMP (misEC^3′U^) (Figures 2A, 3). Interestingly, reaction rates with SAL were not only slower, but became the same in both misEC^3′A^ and misEC^3′U^ (Figure 3), indicating that, in the presence of SAL, reaction lost characteristics of the transcript-assisted hydrolysis, i.e. hydrolysis was not assisted by the 3′NMP of the transcript. This suggests that TL folding is required to reorient 3′NMP for it to be able to assist hydrolysis, and thus accelerate the reaction.

### Distinct properties of sequence-dependently backtracked and misincorporated *E. coli* ECs

The apparent dispensability of the TL in RNA hydrolysis has been observed in ECs with correct 3′ NMP (18). Therefore, we prepared EC that had correct AMP at the 3′ end of the transcript but is naturally stabilized in 1 bp backtracked state due to a specific sequence of the RNA-DNA hybrid (backEC^3′A^; scheme in Figure 2C) (6). This allows minimizing effects of backtracking dynamics on the observed rate of cleavage reaction and, thus, a direct comparison of backEC^3′A^ with misEC^3′A^, stabilized in the same state but through misincorporation.

SAL had much less pronounced inhibitory effect on RNA cleavage in backEC^3′A^; 8.6-fold inhibition compared to 52-fold in misEC^3′A^ (Figure 2, compare B and C). Furthermore, ΔTL RNAP was only 7.5 times slower than WT RNAP in backEC^3′A^, compared to ∼70 times in misEC^3′A^ (Figure 2, compare B and C). These results indicate that, in the backEC^3′A^, the TL involvement in RNA hydrolysis is much more modest than in misEC^3′A^.

Importantly, the rates of the reaction by WT RNAP in misEC^3′A^ and backEC^3′A^ were very close (Figure 2, compare B and C). This suggests that the function of the TL is to convert cleavage-inefficient misEC^3′A^ into a conformation similar to that of backEC^3′A^, which is efficient in cleavage but in which the involvement of the TL in the reaction is modest. Notably, unlike in misEC^3′A^, cleavage by ΔTL RNAP in backEC^3′A^ was slightly inhibited by SAL (∼3-fold). This further supports the idea that the conformation of the 3′NMP of RNA (or other components of the active site that may be involved in transcript-assisted hydrolysis) is somewhat different in misEC^3′A^ and backEC^3′A^, and SAL interferes with it only in the backEC^3′A^.

## DISCUSSION

**Figure 4.**
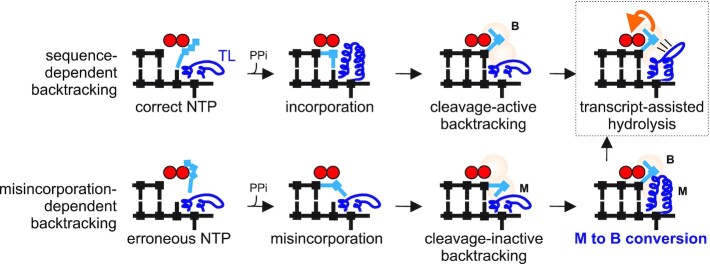
The model of formation of two distinct types of backtracked complexes and role of the TL in conversion of cleavage-inactive into cleavage-active type. Misincorporation (M) and backtracked (B) sites are hypothetical sites that accept 3′ end of RNA during backtracking caused by misincorporation or sequence-dependent backtracking, respectively. 3′NMP in B site is positioned for efficient transcript-assisted catalysis with minimal involvement of the TL in the catalysis. 3′NMP in M site cannot participate in transcript-assisted hydrolysis and requires the TL to shift it into the B site to adopt conformation similar to the one in sequence-dependently backtracked EC. Note that conformation of the TL during ‘M’ to ‘B’ conversion is not known, and the TL folding shown in the cartoon is purely to illustrate the involvement of the TL in this process.

The principal finding of this work is the resolution of a long standing conundrum on the involvement of the TL in RNA hydrolysis by *E. coli* RNAP active center. *Escherichia coli* RNAP was shown to require the TL during RNA hydrolysis in misincorporated ECs (11,13) but appeared not to rely much on the TL during hydrolysis in ECs with correctly paired 3′ end (18). Our results reveal that backtracking of the *E. coli* EC happens through two different ways that lead to distinct conformations of the backtracked complexes with different requirement for the TL for their resolution (Figure 4).

In the correct 1 bp backtracked EC, the 3′NMP of the RNA occupies a site (B site in Figure 4) in the active center from which efficient transcript-assisted hydrolysis of the second phosphodiester bond takes place. In this case, the TL contribution to the cleavage reaction is modest. In contrast, in the EC where backtracking by 1 bp is caused by misincorporation, the erroneous 3′NMP of RNA resides in a conformation incompatible with efficient second phosphodiester bond cleavage (M site in Figure 4). In this case, the role of the TL in cleavage becomes critical, i.e. to reorient the 3′NMP from cleavage-inactive (M) into cleavage-active (B) conformation (Figure 4). The existence of two distinct conformations of backtracked states explains the controversies in the results on the role of the TL in RNA cleavage.

Direct comparison of the misincorporated and correct ECs, both stabilized in 1 bp backtracked state after natural misincorporation and incorporation reveals two pathways of backtracking leading to ECs with different properties. These various paths of backtracking, therefore, suggest that mimicking sequence-dependent backtracking by introducing mismatches at the 3′ end of RNA (usually used in structural analysis of backtracking) may not be valid. Indeed, while RNA cleavage in correct 2 bp backtracked EC was almost independent of the TL (18), cleavage in a similar complexes with 2 mismatched NMPs at the RNA’s 3′ end strongly depended on the TL (11,13), indicating that the latter ECs adopt conformation similar to the misECs, studied here, and cannot be used as a model for sequence-dependent backtracking. Recent structural analysis of *E. coli* EC stabilized in the backtracked state by introduction of four mismatches at the 3′ end of RNA showed that the non-complementary NMP closest to the active center (same as 3'NMP in misEC) would clash with the folded TL, leading to a conclusion that the TL is not involved in RNA hydrolysis (23). However, our data suggest that this NMP resides in the ‘M’ conformation because of its non-complementarity to the template base and needs to be moved to the ‘B’ conformation for hydrolysis to happen. We therefore suggest that the predicted clash of this NMP with the TL, in fact, could reflect the mechanism of the ‘M’ to ‘B’ transition (Figure 4).

Transcript-assisted hydrolysis of phosphodiester bonds of the transcript appears to be conserved at least among bacteria and eukaryotes (3,11,24), pointing at the ancient origin of this phenomenon. The TL is also a very ancient acquisition by RNAP, and, unsurprisingly, appears to have a universal role in phosphodiester bond formation via stabilizing transition state of the reaction (17,25). Surprisingly, however, the role of the TL in RNA cleavage has diverged even within Bacteria. In *E. coli*, the TL participates in conformational transition between two types of backtracked complexes thus activating hydrolysis, but plays modest role in the catalysis *per se*. In *T. aquaticus*, the TL orients 3′NMP, this time in any type of backtracked complexes but also directly participates in general acid-base catalysis (11). Such divergence in functions of the TL during RNA cleavage can be explained by the presence of cleavage factor Gre, which can substitute for the TL in the active center (26) and highly increase the rate of hydrolysis by its own mechanism (27). Interestingly, in cyanobacteria, which lack Gre factors, the rate of TL-dependent intrinsic hydrolysis approaches that of Gre-assisted hydrolysis (12).

Another main finding of our study is the determination of the mode of action of antibiotic SAL. Based on the crystal structure of *E. coli* RNAP holoenzyme in complex with SAL, SAL was proposed to inhibit NMP incorporation by blocking dynamics of the Bridge Helix that was earlier suggested to be essential for the reaction (19). Our results however show that inhibition of NMP addition, pyrophosphorolysis and TL-dependent RNA hydrolysis in misincorporated ECs by SAL is mainly caused by blocking functions of the TL. These results are consistent with structural alignment, which shows that binding of SAL to RNAP creates a steric clash with the folded TL (Figure 1A). We did observe minor (2.5- to 6-fold) inhibition of NMP incorporation by ΔTL RNAP, which may account for the effects of SAL on the TL-independent Bridge Helix functions. We do not know the reason for the discrepancy between our results and the study by Degen *et al.*, (19), who observed similar inhibition by SAL of both, ΔTL and WT RNAPs. One of the reasons could be that we used complete ECs (such as in Figure 1C), compared to a minimal EC containing only downstream portion of the non-template DNA strand used in (19). Lack of non-template strand at the vicinity of the active site may affect translocation properties of the EC and/or arrangement of the reactants in the active site, potentially making TL-independent action of SAL more prominent.
